# Neuroendocrine Carcinoma of the Uterine Cervix: A Clinicopathologic and Immunohistochemical Study with Focus on Novel Markers (Sst2–Sst5)

**DOI:** 10.3390/cancers12051211

**Published:** 2020-05-12

**Authors:** Frediano Inzani, Angela Santoro, Giuseppe Angelico, Angela Feraco, Saveria Spadola, Damiano Arciuolo, Michele Valente, Angela Carlino, Alessia Piermattei, Giulia Scaglione, Giovanni Scambia, Guido Rindi, Gian Franco Zannoni

**Affiliations:** 1 Department of Woman, Child and Public Health Sciences, Gynecopathology and Breast Pathology Unit, Fondazione Policlinico Universitario A. Gemelli IRCCS, 00168 Rome, Italy; frediano.inzani@policlinicogemelli.it (F.I.); angela.santoro@policlinicogemelli.it (A.S.); giuangel86@hotmail.it (G.A.); angefer001@gmail.com (A.F.); saveriaspadola@hotmail.it (S.S.); damiano.arciuolo@policlinicogemelli.it (D.A.); dr.valente.m@gmail.com (M.V.); angela.carlino@policlinicogemelli.it (A.C.); alessia.piermattei@policlinicogemelli.it (A.P.); guido.rindi@unicatt.it (G.R.); 2 ENETS Center of Excellence, Neuroendocrine Tumour (NET) Center, 00168 Rome, Italy; 3 Department of Surgical and Diagnostic Sciences, IRCCS Ospedale Policlinico San Martino, 16100 Genoa, Italy; scaglione.giulia90@gmail.com; 4 Oncological Gynaecology Unit, Department of Woman, Child and Public Health Sciences, Fondazione Policlinico Universitario A. Gemelli IRCCS, 00168 Rome, Italy; giovanni.scambia@unicatt.it; 5 Obstetric and Gynecologic Clinic Institute, Catholic University of Sacred Hearth, 00168 Rome, Italy; 6 Pathological Anatomy Institute, Catholic University of Sacred Hearth, 00168 Rome, Italy

**Keywords:** gynecologic neuroendocrine neoplasms, adenocarcinoma, cervical cancer, immunohistochemistry, somatostatin receptors

## Abstract

Background. Gynecological neuroendocrine neoplasms (NENs) are extremely rare, accounting for 1.2–2.4% of the NENs. The aim of this study was to test cervical NENs for novel markers of potential utility for differential diagnosis and target therapy. Methods. All cases of our center (*n* = 16) were retrieved and tested by immunohistochemistry (IHC) for 12 markers including markers of neuroendocrine differentiation (chromogranin A, synaptophysin, CD56), transcription factors (CDX2 and TTF1), proteins p40, p63, p16INK4a, and p53, somatostatin receptors subtypes (SST2-SST5) and the proliferation marker Ki67 (MIB1). Results. All cases were poorly differentiated neuroendocrine carcinomas (NECs), 10 small cell types (small cell–neuroendocrine carcinomas, SCNECs) and 6 large cell types (large cell–neuroendocrine carcinomas, LCNECs); in 3 cases a predominant associated adenocarcinoma component was observed. Neuroendocrine cancer cells expressed at least 2 of the 3 tested neuroendocrine markers; p16 was intensely expressed in 14 (87.5%) cases; SST5 in 11 (56.25%, score 2–3, in 9 cases); SST2 in 8 (50%, score 2–3 in 8), CDX2 in 8 (50%), TTF1 in 5 (31.25%), and p53 in 1 case (0.06%). P63 and p40 expressions were negative, with the exception of one case that showed moderate expression for p63. Conclusions. P40 is a more useful marker for the differential diagnosis compared to squamous cell carcinoma. Neither CDX2 nor TTF1 expression may help the differential diagnosis versus potential cervical metastasis. P16 expression may suggest a cervical origin of NEC; however, it must be always integrated by clinical and instrumental data. The expression of SST2 and SST5 could support a role for SSAs (Somatostatin Analogues) in the diagnosis and therapy of patients with cervical NECs.

## 1. Introduction

NENs (neuroendocrine neoplasms) represent a wide and heterogeneous family of neoplasms, which are composed of cells with a neuroendocrine phenotype. They may arise anywhere in the body, however, the most frequent sites of occurrence are the gastro-enteropancreatic (GEP) tract and lung [1,2]. These neoplasms show an immunohistochemical profile consistent with neuroendocrine differentiation and may express chromogranin A, synaptophysin, CD56 (N-CAM), PGP9.5, and NSE. These immunomarkers are mandatory for the diagnosis [3].

On rare occasions, NENs may arise in unusual sites such as the female genital tract [4,5,6,7,8]. NENs of the gynecologic tract are extremely rare, accounting for 1.2–2.4% of all the NENs [4,5,6,7,8]. In light of their rarity, histological data consistent with current classification is scant or lacking. 

In this paper we re-evaluated the histological features in a series of cervical NENs diagnosed in our institution and investigated the reactivity to some immunohistochemical markers with the aim to evaluate their usefulness in differential diagnosis and prediction for specific target-therapies with special attention to somatostatin receptors. In detail, we tested a series of immunohistochemical markers, already used in the diagnosis of GEP and pulmonary NENs such as chromogranin A, synaptophysin, CD56 (N-CAM), somatostatin receptors, CDX2, and TTF1. Moreover, p63 and p40, as markers of squamous differentiation, were tested in our series for the differential diagnosis with squamous cell carcinoma of the uterine cervix. Finally, we also analyzed other two markers with potential pathogenic implications: p16, as a surrogate to HPV infection in cervical neoplasms and p53 usually involved in pathogenic mechanisms of poorly differentiated NEC (neuroendocrine carcinoma) of other sites [4,5,6,7,8].

## 2. Results 

### 2.1. Clinical Data 

Paraffin blocks were available in 16 out of the 20 patients retrieved. 

The clinicopathologic features of the 16 investigated cases are summarized in Table 1. The patients’ ages ranged from 30 to 66 years (median 48.5; mean 45). 14 patients underwent surgery (hysterectomy with bilateral salpingo-oophorectomy), with the following distribution for stage FIGO: 2 had disease in stage FIGO IA2, 4 IB1, 1 IB2, 1 IIA2, 3 IIB, 2 IVA, 1 IVB. For the two patients that underwent cervical biopsy, pathological stage was not assessed. 

The observed biological behavior in our case series was the following: 8/16 patients died of disease and 8/16 patients were alive at the moment of the analysis, between them two had a disease relapse.

In detail, we observed 10 metastatic/relapsed patients, with prevalent involvement of the liver and lung. 

Moreover, pelvic and lomboaortic nodal metastases were observed in 5 patients. All data are summarized in Table 1. 

The overall survival (OS) average in dead patients was 24.5 months, while the disease-free survival (DFS) average in patients with recurrences was 13.2 months. 

### 2.2. Pathological Features

**Figure 1:**
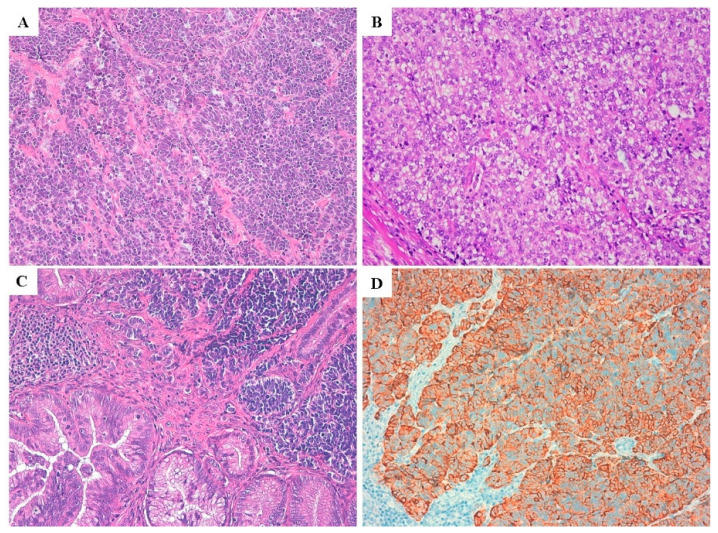
Histological features of small cell type neuroendocrine carcinomas (NEC) of cervix with hyperchromatic nuclei and high nuclear-cytoplasmic ratio (**A**) and large cell type NEC of the cervix with more abundant cytoplasm and focal prominent nucleoli (**B**); foci of AIS may be associated with cervical NEC (**C**); positive immunostain for Chromogranin A is depicted (**D**). (**A**,**B**: 10× magnification); (**C**,**D**: 20× magnification).

**Figure 2:**
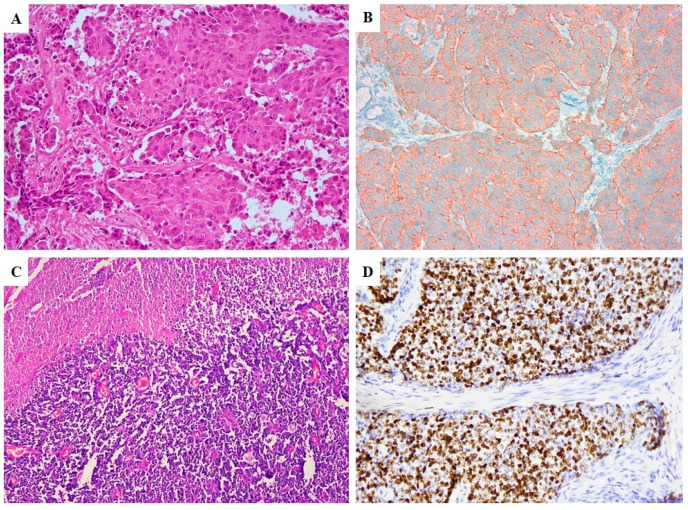
Morphological features of a LCNEC, with abundant eosinophilic cytoplasm (**A**) and diffuse immunohistochemical expression of Synaptofisin (**B**). Morphological features of a SCNEC, with scant cytoplasm, hypercromatic nuclei of small-medium size and geographic type necrosis (**C**) and high proliferative index (Ki67 = 80%) (**D**) (**A**,**B**,**D**: 20× magnification; **C**: 10× magnification).

No well-differentiated NEN was observed. All cases consisted of NEC with small cell morphology in 10 cases (Figure 1A and Figure 2C, small cell–neuroendocrine carcinomas, SCNECs) and large cell morphology in 6 (Figure 1B and Figure 2A, large cell–neuroendocrine carcinomas, LCNECs). All SCNECs displayed a diffuse growth pattern composed of cells with hyperchromatic nuclei, scant cytoplasm, and nuclear molding. LCNECs mostly presented an organoid, nested pattern of growth with an admixed minor component constituted by cord-like structures and solid areas. 

Seven cases also showed an associated non-NE (neuroendocrine) component. In three cases the NE component accounted for 30 to 50% of the tumor cells, associated with invasive adenocarcinoma and the tumors were defined as mixed neuroendocrine–non neuroendocrine neoplasms (MiNENs) [6,7,8]. In four cases the non-NE component represented only focal areas (approximately <5%) of the entire neoplasm and consisted of: high grade squamous intraepithelial lesion (H-SIL/CIN3), adenocarcinoma in situ (AIS) (Figure 1C) and invasive endocervical adenocarcinoma. 

The mitotic activity of NEC was brisk, with an index ranged from 12 to 65 mitoses/10 HPFs (mean 27.57). Lympho-vascular space invasion (LVSI) was present in eight cases. The mitotic activity of the adenocarcinoma component of MiNENs was evidently lower ranging from 7 to 25 mitoses/10 HPFs (mean 12.75 mitoses/10 HPFs).

### 2.3. Immunoprofile 

**Figure 3:**
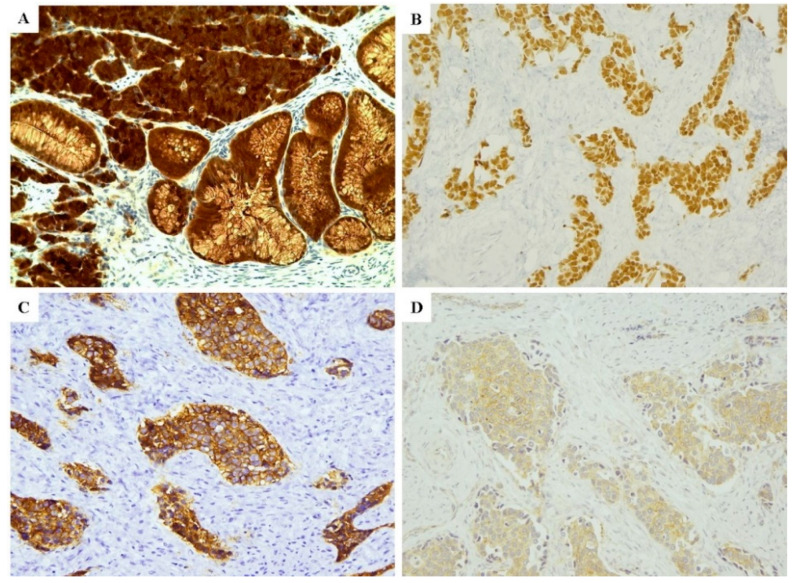
Immunohistochemistry for p16 usually shows diffuse positivity in cervical NEC (**A**); CDX2 is often well expressed in our case series (**B**). Two representative examples of expression of somatostatin receptors: score 3 for SST2 (**C**) and score 2 for SST5 (**D**). (**A**–**D**: 20× magnification).

The immunohistochemical scores of the NE components are detailed in Table 2. All NECs, either pure or mixed, showed expression of at least two neuroendocrine markers (Figure 1D and Figure 2B). Ki67 proliferative index showed high values ranging from 45 to 98% (median of 87.5%) (Figure 3B). 

p63 and p40 expressions, characteristic for the squamous histological type, were negative, with the exception of one case that showed moderate expression for p63. p16INK4a was intensely expressed in 14 cases (87.5%) (Figure 3A), and possibly related to HPV infection; in the remaining two cases it was negative or only moderately (patchy) expressed. In this regard, it is well known that cervical NECs are related to HPV infection and p16 represents a reliable surrogate marker for the infection. Therefore, since HPV test was not available in our institution, p16 immunohistochemical expression served as a surrogate marker to the infection [4,5,6,7,8]. Five cases (31.25%) presented positivity to TTF1, two LCNECs with faint and focal intensity (+1), three SCNECs with mild intensity (+2). Eight cases (50%) were positive for CDX2 (Figure 3B); of these, two were SCNECs with moderate intensity (+2), six LCNECs with moderate-high positivity (+2, +3) in all except one which displayed low positivity (+1). Among eight negative cases, seven were NECs of small cell type and one of large cell. 

SST2 was positive in 8 cases out of 16 (50%) (Figure 2C and Figure 3C), with score ranging from 2 to 3; of these, five out of eight cases (62.5%) were SCNECs. SST5 expression was observed in 11 out of 16 patients, with scores ranging from 2 to 3 in nine cases (two cases with score 1, six with score 2, and one case with score 3) (Figure 3D). 

As for the three cases with a minor NE component, the adenocarcinomatous cells showed an immunoprofile analogous to NE cells, with negativity for p63, p40, and TFF1 and only focal staining for p53 in all cases; while p16 was positive in all three cases. Differently from NE cells, the adenocarcinomatous cells were consistently negative for chromogranin A, synaptophysin, CD56, SST2, and SST5. Similar to the NE component, CDX2 was also positive in the adenocarcinoma component in two cases. 

### 2.4. Statistical Analysis 

The association between prognosis of patients (alive vs. dead), clinicopathological and immunohistochemical features did not show statistically significant results. 

## 3. Discussion 

NENs of the gynecological tract are very rare; the uterine cervix represents the most common site and poorly differentiated NEC is the most represented type [6,7,8,9]. In this case series, we examined a series of 16 cervical NECs, both small and large cells types, which were diagnosed in our institution between 2007 and 2017. We tested immunohistochemical markers of potential usefulness for the differential diagnosis, for prognosis assessment and to predict responses to specific target-therapies. 

The morphological distinction between cervical NEC and poorly differentiated squamous cell carcinoma represents a major diagnostic challenge. 

In this context, immunohistochemistry (IHC) for p63 and p40 may be useful since these markers are consistently expressed in squamous cell carcinomas. In contrast, in our series of histologically confirmed cervical NECs, 15/16 cases showed negative stain for both antibodies, while only one case (SCNEC) showed moderate immuno-expression for p63. Our data support the use of p63 and p40 for the diagnosis of cervical squamous cell carcinoma rather than NEC as previously proposed by Wang et al. [10]. In particular, while p63 expression was also reported in a non-negligible percentage of cervical NECs [11], p40 excludes their diagnosis. Therefore, p40 appears as a more reliable marker for squamous differentiation because when diffusely expressed, it rules out the possibility of a glandular or neuroendocrine differentiation; moreover it is consistently negative in NENs at any site [12,13].

Before rendering a diagnosis of cervical NEC, the possibility of a metastatic nature from other sites, mainly GEP and lung, must be ruled out. Unfortunately, in our study, IHC has proven to have limited usefulness regarding markers as TTF1 and CDX2 in this type of differential diagnosis. In fact, TTF1 was positive in a discrete amount of the present series (5 cases, 31.25%) and literature data report its frequent expression in NEC at various extrapulmonary primary sites [10,11,14]. CDX2, a reliable marker to establish the neoplastic origin from GEP [15], was significantly expressed in our series (8/16 cases showed positive stain). Interestingly, CDX2 positivity in cervical NEC is a novel finding, never reported in literature, and it may be further explored in future studies. Moreover, CDX2 is expressed in another rare histotype of cervical cancer: intestinal type cervical adenocarcinoma [16,17].

According to our findings, the identification of a synchronous cervical neoplasia (either intraepithelial or invasive), in close proximity to the neuroendocrine tumor, is strongly suggestive for the cervical origin of NEC. In this regard in our series, we observed foci of intraepithelial cervical neoplasia in four cases (2 H-SIL and 2 AIS) and foci of invasive cervical adenocarcinoma of usual type were detected in four cases.

Based on our results, the observed frequent expression of CDX2 and TTF1 and the negativity for p63 and p40 may suggest that the pathogenesis and differentiation line of cervical NEC is closely related to cervical glandular neoplasms.

As widely described in literature, cervical NEC is related to HPV infection, and mainly in high-risk HPV 16 and 18 [18,19]. These neoplasms usually show the intense immunohistochemical expression of p16 as a surrogate marker to infection [11,18].

Most of our cases were positive for p16. Recently, HPV infection has been also demonstrated in MiNEN of the large intestine [20]. On the other hand, we have to keep in mind that p16 immunoreactivity may also be observed in not HPV-related neoplasms as high grade serous carcinomas or leiomyosarcomas among gynecological malignancies, and poorly differentiated NENs of other sites [21,22,23].

All studied neoplasms showed high Ki67 proliferative index, with values ranging from 45% to 98% (median of 87.5%).

Among the GEP-NENs Ki67 has taken on a key role in grading these neoplasms and therefore in their prognostic evaluation. 

In the uterine cervix, due to the rarity of NENs at this site, precise reference values for Mib1/Ki67, useful to a prognostic classification have not yet been identified.

In this regard, in our series, the association between prognosis of patients (alive vs. dead), clinicopathological features, and immunoprofile (also including Ki67 expression) did not show statistically significant results. Multicentric studies on large cases, comprising also well differentiated NENs are necessary for this purpose. 

The nuclear over-expression of p53, observed by IHC in NECs of the lung and GEP tract, reflects the frequent mutations in the onco-suppressor gene *TP53* in these neoplasms [24]. However, in our series of cervical NECs, no cases showed p53 over-expression by IHC, suggesting that *TP53* may not have a pathogenetic role in these neoplasms. 

The most interesting data were finally related to SST2 and SST5. In the present series, SST2 and SST5 showed positive staining, with high scores in half of the cases. The present paper represents the largest case series available on cervical NECs. 

Only few previous studies have reported data regarding SST2 and SST5 expression in cervical NECs. SST2 was reported positive at IHC in 3 of 7 NECs [25]. Additionally, 68Ga-DOTATATE PET/CT was reported positive in vivo in a cervical SCNEC [26]. Two cervical NECs, one pure, the other combined with a squamous cell carcinoma, showed low expression of SST2-5 by Real Time RT-PCR with only weak stain at IHC [27]. Overall, these data may support the use of SSAs (Somatostatin Analogues) in vivo for the diagnosis and the therapy of cervical NEC with both hot and cold analogues [28]. Further studies are needed to verify the prognostic and predictive value of the immunohistochemical expression of SST2 and SST 5 in patients with cervical NEC. 

## 4. Methods 

### 4.1. Ethic Statement and Patient Selection

Twenty cases of NEN arising in the uterine cervix were retrieved from the files at our institution (Pathology Unit, Catholic University of Sacred Hearth, Rome) covering a 10-year period (2007–2017). All patients performed their primary surgery at our hospital. 

Our study was conducted in accordance with Good Clinical Practice guidelines and the Declaration of Helsinki (1975, revised in 2013). The clinical information had been retrieved from the patients’ medical records and pathology reports. Patients’ initials or other personal identifiers did not appear in any image. Finally, all samples were anonymized before histology and immunohistochemistry. The Institutional Review Board (IRB) of Fondazione Policlinico A. Gemelli—IRCCS, considered the retrospective nature of the study and approved the submission of our scientific work (N. Prot. 19640/20). 

Analyzed data were collected as part of routine diagnosis. Patients were diagnosed and treated according to national guidelines and agreements. Our analysis looked retrospectively at treated patients’ outcomes. This was done internally as part of an audit/evaluation, so as to improve our quality of care.

### 4.2. Pathological Assessment 

Pathology reports, hematoxylin-and-eosin (H&E), and immunohistochemical stained slides were reviewed by two expert pathologists (GFZ, FI), in order to confirm the initial diagnosis. Moreover, the following pathological variables were recorded: tumor size, histotype, LVSI, depth of cervical stromal involvement, resection margins status. Clinical information, including patients’ ages, treatment, tumor stage, and follow-up were retrieved from the patients’ charts and from their treating physicians. 

The pathological diagnoses of SCNEC, LCNEC, and MiNEN were made in accordance to the WHO criteria for neuroendocrine tumors of the uterine cervix, also following the unitarian IARC-WHO proposal [29,30].

### 4.3. Immunohistochemistry 

IHC was performed using the Ventana automated immunostainer (Ventana Medical Systems, Tucson, AZ, USA). The following tests were performed: p16 (E64H, 1:3 of pre-dilute, Ventana, Tucson, AZ, USA), TTF-1 (8G7G3/1, 1:200, Dako); Synaptophysin (MRQ-40 Rabbit monoclonal, pre-dilute, Roche Ventana Medical Systems, Inc., Tucson, AZ, USA), anti-chromogranin A (LK2H10, pre-dilute, Roche Ventana Medical Systems), anti-CD56 (Clone 123C3, pre-dilute, Dako, Denmark A/S); anti-p53 (DO-7, pre-dilute, Dako, Denmark A/S), anti-p40 (BC28, 1:50, Biocare, Pacheco, CA, USA), anti-p63 (4A4, pre-dilute, Roche, Ventana Medical Systems, Inc., Tucson, AZ, USA), anti-Ki67 (MIB1, pre-dilute, Dako, Denmark A/S), Anti-Somatostatin Receptor 2A (UMB1, 1:5000, Abcam, USA), Anti-Somatostatin Receptor 5 (UMB1, 1:5000, Abcam, USA) and anti-Human CDX2 (EPR2764Y Rabbit Monoclonal, pre-diluted, Roche Ventana Medical System, Inc. Tucson, AZ, USA). 

With the exception of p53 and SST2-5, IHC results were scored as follows: 0: negative (no cells stained); 1: focal positive (≤10% cells stained); 2: patchy (11% to 49% cells stained) and 3: diffusely positive (≥50% of cells stained). For p53, the scores were: negative (no cells stained), 1: focal positive (≤10% cells stained); 2: patchy (11% to 74% cells stained) and 3: diffusely positive (≥75% of cells stained) [31]. Scores 0 and 3 were considered as potentially mutant (all or null staining); scores 1–2 were considered as a wild-type. SST2 and SST5 were scored according to Volante et al. [32] as follows: 0, absence of stain; 1, only cytoplasmic immunoreactivity, focal or diffuse; 2, membranous reactivity in <50% of tumor cells, irrespective of the presence of cytoplasmic staining; 3, circumferential membranous reactivity in >50% of tumor cells, irrespective of the presence of cytoplasmic staining. Cases with a score of 2–3 were considered as positive, and 0–1 were considered as negative. 

### 4.4. Statistical Analysis 

The association between prognosis of patients (alive vs. dead), clinicopathological and immunohistochemical features was assessed by a regression logistic text. Only *p* < 0.05 was considered as significant result. 

## 5. Conclusions 

In our case series, the observed frequent expression of CDX2 and TTF1 in NENs of the cervix and conversely the negativity for squamous differentiation markers (p63 and p40) may suggest that the pathogenesis and differentiation line of cervical NEC is more closely related to cervical glandular neoplasms than squamous. By the way, IHC has proven limited usefulness regarding markers as TTF1 and CDX2, which can be expressed by a NEN of the cervix as well as by a metastatic NEN from lung or GEP tract. 

Immunohistochemical p16 expression may suggest a cervical origin of NEC; nevertheless, in order to establish the definitive cervical primitivity, we must integrate morphological and immunophenotypical findings with correct clinical, anamnestic, and radiological data. Moreover, the histological evidence of other in situ or invasive cervical lesions (i.e., HSIL, AIS) may further support the cervical origin of the tumor.

Finally, SST2 and SST5 appear consistently expressed in cervical NECs. It follows that overall, these data may suggest a potential of SSAs in vivo for the diagnosis and the therapy of cervical NEC with both hot and cold analogues [28]. Further clinical-pathological studies are needed to verify the possible role of SSAs in the management of cervical NEN and to assess the prognostic and predictive value of the immunohistochemical expression of SST2 and SST5 in this context.

## Figures and Tables

**Table 1:** Clinicopathological data.

Cases	Age (ys)	Surgical Procedure	Histotype	FIGO Stage	Associated Neoplastic non-NE Component	Prognosis	Recurrent/Metastatic Disease (Site)	Follow-Up (Months)	Treatment
1	35	HA	SCNEC	IA2	-	DOD	Pancreas	36	Cysplatin + Etoposide (7 cycles) + BRT
2	36	HA	SCNEC	IA2	-	A		36	Cysplatin + Etoposide (6 cycles) + BRT
3	45	HA	LCNEC	IB1	HSIL	A		24	Cysplatin + Etoposide (6 cycles) + BRT
4	41	HA	LCNEC + IA	IB1	-	AWD	Lung, bone, liver, pelvic limph nodes	30	NAD: Cysplatin + Etoposide (6 cycles) AD: Cysplatin (1st line); Topotecan + Etoposide (2nd line) + BRT
5	30	HA	LCNEC	IB1	HSIL, AIS	A		24	Cysplatin + Etoposide (5 cycles) + BRT
6	81	HA	LCNEC + IA	IB1	-	DOD	Peritoneal carcinosis, liver metastases	28	Cysplatin + Etoposide (6 cycles) + BRT
7	44	HA	SCNEC + IA	IB2	-	A		46	Cysplatin + Etoposide (6 cycles) + BRT
8	57	HA	SCNEC	IIA2	-	DOD	Lung, lombo-aortic lymph nodes	6	Untreated
9	33	HA	SCNEC	IIB	-	AWD	Subcutaneous and pelvic recurrences	28	1st line: Carboplatin + Etoposide (7 cycles) + BRT 2nd line: Topotecan + Etoposide
10	50	HA	SCNEC	IIB	-	DOD	Liver, pelvic lymph nodes	48	Cysplatin + Etoposide (6 cycles) + BRT
11	50	HA	SCNEC	IIB	-	DOD	Lung, liver, pelvic lymph nodes	26	Cysplatin + Etoposide (4 cycles) + BRT
12	62	HA	LCNEC	IVA	-	DOD	Liver	10	Cysplatin + Etoposide (6 cycles) + BRT
13	62	HA	SCNEC	IVA	-	A		60	Cysplatin + Etoposide (4 cycles) + BRT
14	66	HA	LCNEC + IA	IVB	-	DOD	Peritoneal carcinosis, lung metastases	18	Cysplatin + Etoposide (3 cycles) + BRT; After tumoral progression: Taxol + Bevacizumab + Gemcitabin
15	61	B	SCNEC	ND	-	DOD	Bone, lombo-aortic lymph nodes	24	Cysplatin + Etoposide (6 cycles) + BRT
16	47	B	SCNEC	ND	AIS	A		36	NAD: Cysplatin + Etoposide (3 cycles) AD Cysplatin+Etoposide (7 cycles) + BRT

HA: Hystero-annessiectomy; B: Biopsy; LCNEC: Large Cell Neuroendocrine Carcinoma; SCNEC: Small Cell Neuroendocrine Carcinoma; IA: Invasive Adenocarcinoma; HSIL: High Grade Squamous Intraepithelial Lesion; AIS: Adenocarcinoma in situ; A: alive without recurrent disease; DOD: dead of disease; AWD: alive with recurrent disease; NAD: adjuvant therapy; AD: adjuvant therapy; BRT: brachytherapy.

**Table 2:** Immunohistochemical findings.

Cases	FIGO Stage	CgA	Syn	CD56	TTF1	CDX2	p16	p53	p40	p63	SSTR2A	SSTR5	Ki67/Mib1
1	IA2	3	3	3	0	0	3	1	0	0	1	0	90%
2	IA2	2	3	3	2	2	3	1	0	2	2	2	98%
3	IB1	3	3	0	1	1	3	1	0	0	0	0	70%
4	IB1	3	3	2	1	3	3	1	0	0	0	2	60%
5	IB1	3	3	3	0	3	3	1	0	0	3	2	95%
6	IB1	2	3	3	0	2	0	1	0	0	0	0	45%
7	IB2	3	3	0	0	3	3	1	0	0	2	2	80%
8	IIA2	2	3	2	0	0	3	3	0	0	2	1	90%
9	IIB	3	3	3	0	0	3	1	0	0	2	0	95%
10	IIB	3	3	3	2	2	3	1	0	0	3	2	80%
11	IIB	2	2	2	0	0	3	1	0	0	0	2	90%
12	IVA	3	2	3	0	0	3	1	0	0	0	2	75%
13	IVA	0	2	3	0	0	2	1	0	0	0	0	80%
14	IVB	3	3	2	0	2	3	1	0	0	3	3	85%
15	ND	2	3	3	0	0	3	1	0	0	0	2	95%
16	ND	3	3	3	2	0	3	1	0	0	2	1	95%

ND: not determined.
